# PPE decontamination to overcome PPE shortage in rural area during pandemic

**DOI:** 10.1016/j.infpip.2021.100145

**Published:** 2021-04-29

**Authors:** Abdurrahman Kharbat, Habib Abla, Mahmud Alkul, Ranger Kile, Justin White, Cynthia Reinoso Webb, Steven M. Presley, Min H. Kang

**Affiliations:** a School of Medicine, School of Medicine, Texas Tech University Health Sciences Center, Lubbock, TX, 79430, USA; b Graduate School of Biomedical Sciences, School of Medicine, Texas Tech University Health Sciences Center, Lubbock, TX, 79430, USA; c Office of President, School of Medicine, Texas Tech University Health Sciences Center, Lubbock, TX, 79430, USA; d Department of Pediatrics, School of Medicine, Texas Tech University Health Sciences Center, Lubbock, TX, 79430, USA; e Department of Environmental Toxicology, The Institute of Environmental and Human Health, Texas Tech University, Lubbock, TX, 79416, USA

**Keywords:** COVID-19, Personal protective equipment, N95 respirator, Hydrogen peroxide vaporization

## Abstract

Despite remarkable developments in healthcare, the world was not ready to stop the spread of the novel COVID-19 pandemic almost a century after the great influenza pandemic. The explosive increase in the number of patients stalled the healthcare system, and the first and apparent issue was the shortage of personal protective equipment (PPE). Our group established a system using a hydrogen peroxide vaporization method to decontaminate and reuse N95 respirators for healthcare workers. The system decontaminated over 12,000 units of PPE to cover institutions in West Texas. This service provided support at the most needed time during the pandemic.

## Introduction

As severe acute respiratory syndrome coronavirus 2 (SARS-CoV-2) spread across the world, and the COVID-19 pandemic was set in its course, governments and healthcare systems around the world were overcome by the burden of a novel pathogen for which we were unprepared. Through a series of unfortunate events, COVID-19 left healthcare systems with not only a high mortality count but also an unprecedented depletion of healthcare resources [1]. Chief amongst the resources ravaged by the pandemic on a global scale was personal protective equipment (PPE), which left physicians and other healthcare professionals at an increased risk of contracting SARS-CoV-2 and developing COVID-19 [2]. As the pandemic progressed, international and even national cooperation decreased, and various states within the United States were instructed to acquire and manage their own reserves of PPE [3]. With COVID-19 infection rates increasing in the State of Texas and the mortality count increasing in its wake, various municipalities looked for opportunities to take ownership of their reserves of PPE. As of the beginning of January 2021, there have been more than 3 million cases and 43 thousand fatalities in the State of Texas (https://txdshs.maps.arcgis.com/apps/opsdashboard/index.html#/ed483ecd702b4298ab01e8b9cafc8b83). To this end, the Texas Tech University Health Sciences Center (TTUHSC), which serves the vast West Texas and South Plains regions, set out to develop a process that would allow rural municipalities to bear the burden of PPE depletion caused by the COVID-19 pandemic.

With the unprecedented global depletion of PPE resources came a push to identify and develop processes for PPE decontamination. After the rigorous reviews of proposed decontamination methods and the available resources [[4], [5], [6], [7], [8]], our institution selected hydrogen peroxide decontamination through vaporization as the process that could most effectively and safely decontaminate large amounts of PPE, specifically filtering face-piece respirators (FFRs) and face-shields, whilst maintaining the functional performance of the decontaminated PPE [9]. The Food and Drug Administration (FDA) reinforced the efficacy of the hydrogen peroxide vapor process through an Emergency Use Authorization (EUA), which allowed institutions to reuse PPE that was decontaminated with hydrogen peroxide vapor. This process utilizes the Bioquell Z Vapor Hydrogn Peroxide Generator (VHP), which is able to safely and effectively vaporize hydrogen peroxide within a sealed environment, killing all pathogens [4,5]. TTUHSC working with The Institute of Environmental and Human Health (TIEHH), Texas Tech University developed such a system, which contained enough space to safely decontaminate 10,000 units of PPE at a time. Effective decontamination was confirmed through commercial biological indicators inoculated with *Bacillus* bacteria, which when subjected to the VHP decontamination yielded a negative reading.

## Methods

### Collection and redistribution of PPE

Healthcare organizations, nursing homes, and dental offices utilizing the decontamination service provided by TTUHSC were instructed to double bag their used and non-visibly soiled N95 FFRs, surgical masks, face shields, and safety glasses and place them in biohazard waste containers labelled with the entity's name or floor number as well as the initials of the users. Contaminated PPE containers were collected on Monday and Thursday mornings and delivered to the decontamination site. The containers were marked with site name and each PPE was marked with the user name on the strap so that the PPE would return to the original user to minimize the fitting issue. Biohazard bins and PPE were decontaminated overnight and collected Tuesday and Friday mornings. Sterile PPE was redistributed upon collection of additional contaminated PPE the following Monday and Thursday, allowing for two decontamination cycles per week. Appropriately trained and certified TIEHH personnel operated the Bioquell VHP generator.

### Decontamination site lay-out and PPE placement

A standard 20-foot shipping container with exterior dimensions of 20′(L) x 8′6”(W) x 8′(H) was lined with ten stainless steel racks containing seven shelves each. Individual shelves were assigned to and labelled with entity names according to the volume of PPE they provided. Groups of PPE exceeding 24 items per entity required multiple shelves. A Bioquell Z Vapor Hydrogen Peroxide Generator was placed in the central clearing of the shipping container along with a standard house fan to increase air circulation. Appropriate PPE consisting of an N95 FFR, safety goggles, safety gown, and both nitrile and latex gloves were donned by team members prior to accessing contaminated materials. N95 FFRs and other PPE were then placed on the shelves in an organization of three rows by eight columns. Biohazard bins were propped against the racks, ensuring that the lid remained open. Eight APEX® Disc biological indicators (MesaLabs, catalog number: GRS-090, population 2.4 × 10^6^) were used to monitor the efficacy of VHP cycles. *Bacillus atrophaeus* packaged in a medical grade Tyvek pouch, which is permeable to VHP were distributed throughout the shipping container, at high, medium, and low levels. Some biological indicators were placed underneath PPE to ensure decontamination occurred on both sides. Upon completion of the decontamination cycle, the biological indicators were collected and placed in sterile Triptic Soy Broth (TSB) and incubatted alongside a positive and negative controls. Before returning them to the users, the decontaminated batch of PPE was held until the results of the surrogate bioindicators were available.

### Decontamination protocol and indicator analysis

The VHP decontamination protocol developed by Brooks *et al.* to ensure 6-log reduction in organism viability using the Bioquell system was adopted [6]. This 4-phase, 8-hour protocol consisted of conditioning, gassing, dwelling, and aerating. A 10-minute conditioning phase prepared the VHP for gassing the container and assessed the temperature and room humidity conditions. During the gasing phase, hydrogen peroxide was injected at a rate of 10 grams per minute with the goal of creating microcondensation of hydrogen peroxide as quickly as possible to reach a peak concentration based on the room temperature an danbient humidity. Once microcondensation of hydrogen peroxide was established at average 625.53 ppm, a 25 minute dwelling phase began and maintained a plateau of the hydrogen peroxide concentration. Once the dwell time concluded, the aeration phase allowed hydrogen peroxide levels to fall to undetectable levels via catalyzation into water and oxygen. The process was carried out at a temperature range of 28–40 °C. Following cycle completion, the biological indicators were incubated alongside positive and negative controls. Negative control was treated with 10% bleach solution for 30 minutes, then placed in a TSB tube. Positive control was not treated and placed directly in a TSB tube. All tubes were incubated at 35°C for 7 days. Tubes were assessed for bacterial growth daily by observing for turbidity. Lack of turbidity or sediment indicated no growth in the negative control and test biological indicators, thus a successful decontamination process through VHP. Positive control had bacterial growth demostrated by turbidity and sediment accumuation. The test tubes were reviewed for seven days before respirators were cleared to be returned to the users.

## Results & discussion

**Figure 1:**
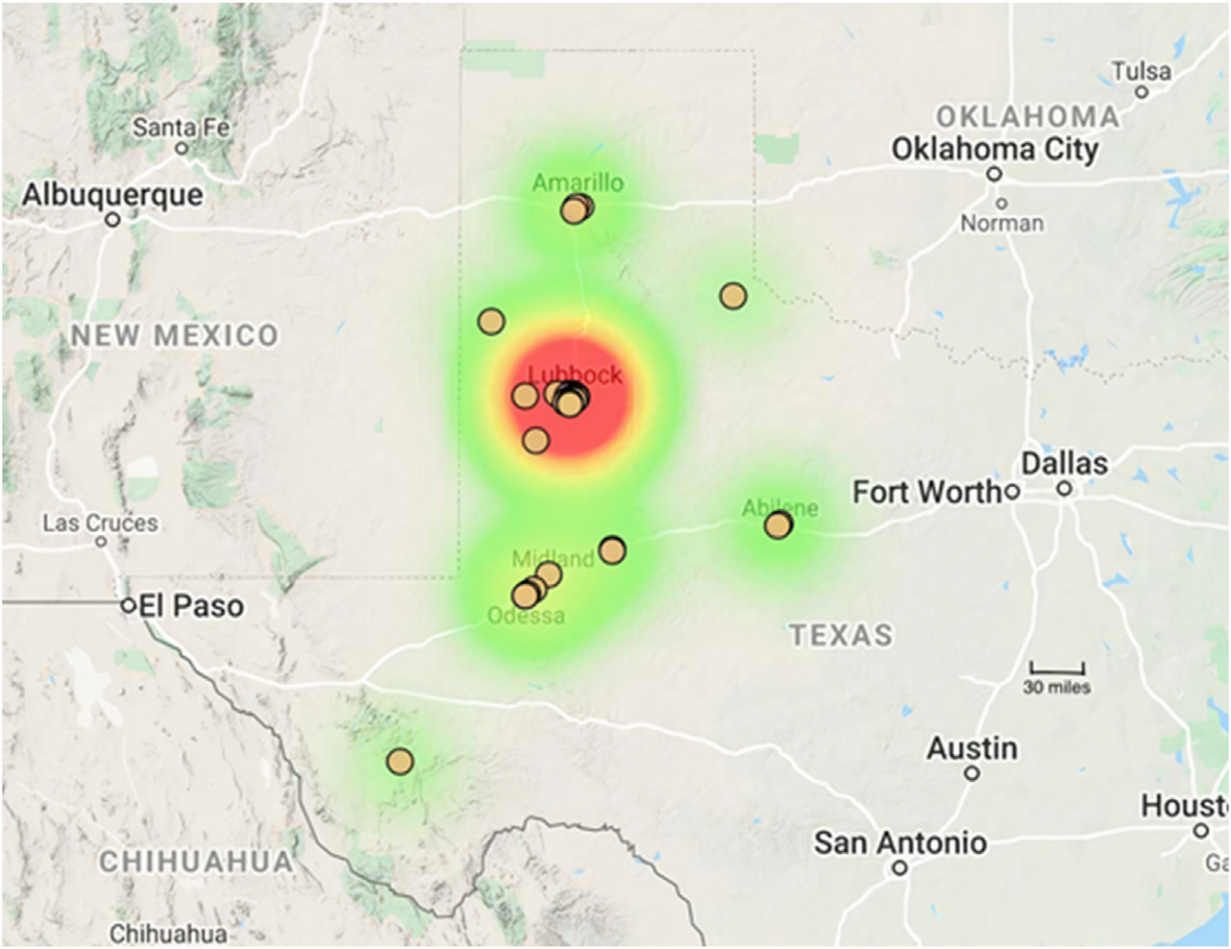
TTUHSC PPE Decontamination Service Utilization Heatmap for the South Plains and West Texas Region. Yellow dots represent each healthcare organization or clinic that used N95 decontamination service. Red to green: higher to lower number of N95 FFR's decontaminated.

**Figure 2:**
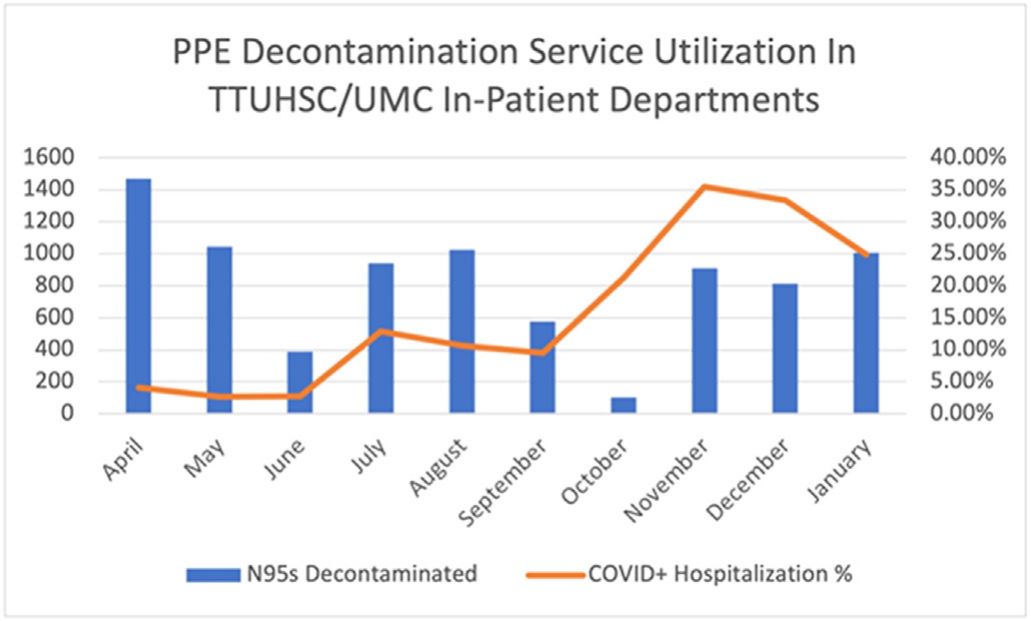
PPE Decontamination Service Utilization relative to the number of COVID-19-associated hospitalization in TTUHSC/UMC In-Patient Departments.

Our PPE decontamination service was active from April 16^th^, 2020 to February 24^th^, 2021. Over the time period, more than 12,000 N95 FFRs were decontaminated, with 9,083 coming from TTUHSC and University Medical Center in-patient departments, ranging from Medical Intensive Care Unit to Emergency Center. We provided the free-of-charge decontamination service to 56 healthcare organizations and clinics in Texas, and the heatmap qualitatively and quantitatively presents the efficacy of our service per region (Figure 1). Also, a chart details service utilization rates plotted against the percentage of patient hospitalizations that are COVID-19 positive (Figure 2).

It is abundantly clear (Figure 1) that our service was able to significantly ameliorate the PPE shortage that struck our region and continues to serve our healthcare workers to this day. Moreover, an examination of the PPE decontamination service utilization by TTUHSC/University Medical Center in-patient departments (measured by the number of N95 FFRs delivered for decontamination per month) plotted against the percentage of hospitalized patients who were COVID-19 positive (Figure 2) shows that in the months of April through September, the rates of service utilization closely followed the percentage of COVID-19 positive hospitalized patients. In the month of October, a transient increase in the supply of PPE to our region resulted in our process shifting to once a month PPE decontamination. This increase in PPE supply led to a decrease in our service's utilization for the month of October. Most notably, however, as COVID-19 positivity rates of hospitalization increased, albeit an increased supply of PPE, our service's utilization rates picked up significantly, demonstrating efficacy of our process and further reinforcing our contention that such a PPE decontamination service is integral to the resiliency of a hospital system's fight against a resource draining pandemic like COVID-19.

The importance of establishing a self-sufficient and self-sustaining healthcare system in the context of pandemic planning cannot be overstated. Our novel undertaking, which paved the way for our region to effectively combat the COVID-19 pandemic, has demonstrated efficacy in the preservation of PPE resources. By providing this PPE decontamination service for our region's hospitals and clinics at no cost, we were able to mitigate the risk of PPE procurement issues in numerous rural municipalities. Moreover, this initiative highlighted the importance of being able to quickly respond to shortages in PPE and potentially prevent the spread of the infection in healthcare workers. The establishment of a regional decontamination initiative served West Texas and the South Plains regions well, and should be considered as one of the contributory factors in overcoming the unprecedented pandemic around the world.

## Credit author statement

AK, HA, AM, RK, JW, and MHK collected, decontaminated, and re-distributed PPE's from and to the users. MHK reviewed and selected the method of decontamination. JW and MHK created the network for the PPE decontamination. CRW and SMP provided the logistics and operated the decontamination process at TIEHH. AK and MHK wrote the paper.
